# Present-Day Problems

**Published:** 1888-12-15

**Authors:** 


### THE DOCTOR'S PULPIT.

*(Continued from page 150.)*

### PRESENT-DAY PROBLEMS.

### SOCIAL PARASITES.

PARASITISM is a curious and instructive study. Everybody is familiar with the fact that

"Great fleas have little fleas
Upon their backs to bite 'em;
Little fleas have lesser fleas,
And so *ad infinitum*."

But why should it be so? Of what mortal use is a flea, or a bug, or a "pediculus"—the English word "louse" has gone out of fashion? Nevertheless, whether these creatures be of any use or not, there they are in infinite variety, on the skin, in the skin, in the bowel, in the liver, here and there and everywhere, filthy and tormenting fiends. One might have thought that at least the creatures would have belonged to classes and orders of their own; that they would not have been able to claim any *bonâ fide* kinship with respectable and well-conducted animals. But that is by no means the case. The greedy thieves that lurk in the alimentary canal are the blood relations of all the other *vermes*; and the prowling vagabonds that wander up and down the head, or burrow in the skin, belong to the large, respectable, and often beautiful order of the *insecta*. It is a mystery, neither greater nor less than a thousand other mysteries, that exercise the ingenuity and try the patience of "wondering and hard-driven man."

It is a well-known law in the animal world that certain forms of parasitism are in the direct ratio of physical feebleness and debility. A sheep in poor health will be covered with vermin, whilst a robuster "mutton" will hardly be troubled at all. The skin of a lean and hungry cow is a burrow for numbers of huge, fat, maggot-like creatures which one would think the animal could not possibly survive; but the healthier integument of a plump shorthorn may be almost completely free. Nature in some of her moods seems both wilful and cruel. Perhaps she is not! But we see all kinds of greedy creatures ready in a moment to rush down with gluttonous maw upon any animal which shows the least sign of feebleness or approaching dissolution. The presence of many parasites is both an index and an accompaniment of feebleness and bad health.

Is there any analogy between these circumstances and the life of the community? Undoubtedly; the clearest analogy. Following the lead of Mr. Herbert Spencer, we may be guided to distinct vision and to practical conclusions of the first importance. It is essential to understand at the outset, and to admit freely to ourselves, that a parasite *is* a parasite. Last week we showed that, tried by certain standards, half the population of London, and nearly the same proportion in the country, are "inferior" or "wasters." Most of the "wasters," and many of the "inferior" classes, are "parasites." These may seem hard words to use of human beings, but the words are not harder than the deeds of Nature. She leaves "inferior" persons to lack many things, whilst "wasters" she allows to starve. Some parasites merely irritate the hosts on which they live; others suck the life-blood. It is precisely so with the different social parasites which infest the community. Occasional paupers are of the irritating class. Inbred and confirmed paupers, of which there are many, and inbred and confirmed thieves, suck the life-blood of the people on whom they prey. Pity for paupers and thieves is a fine sentiment: and a brotherly desire to help is worthy of all praise. Of these we shall have more to say at a later stage of this discussion. At present, our mood should be severely scientific: we must look the facts squarely in the face. An "inbred" pauper is not a meritorious person. He does not deserve pity. He is a pauper, not because his body is weak, not because his mind is weak: but because he is vicious. It is the intention of Nature that he shall starve if he will not work. Starvation is Nature's whip by which she drives the lazy to their tasks. It is a very proper and a very necessary whip. As St. Paul, with robust good sense says, "If any man will not work neither shall he eat;" and in another place, "He that neglecteth to provide for his own . . . . hath denied the faith, and is worse than an Infidel." Those who pity the poor with indiscriminate pity, and those who help with a universal imbecility of good nature are not the friends but the enemies both of the poor and the rich. "It is so hard," say they, "to know who are deserving and who are not; and it is better to help ten undeserving than that one deserving should go unhelped." Perhaps! On no account should the deserving be unhelped. But if the deserving would exercise a little full-grown judgment, and not run about recklessly from place to place, they would not so readily get mixed up with the undeserving. A rich man, or a middle-class man like a parson or a doctor, cannot afford to throw up his work at a day's notice and seek a new home elsewhere. He must wait, if he wishes to change, and make his plans with judgment, and then bear the consequences. It is often thought that if a poor man be honest and industrious he must be "deserving." He may be, or he may not be. He may be an honest and industrious fool, or a hot-tempered, impetuous fellow. He may throw himself out of work without reason. But Nature says he shall suffer for these defects of character. Her intention is to make men not merely honest and industrious, but sensible, reasonable. She has no patience with grown-up babies. In the period of real infancy she provides for them. She fills parents' hearts with a measure of love sufficient to ensure the adequate care of the infant. But when human beings are of full age, whatever be their position or education, Nature demands of them reasonableness, good sense, and capacity, as well as honesty and industry. If they display these qualities their success is generally assured. If they show a lack of any or all of them their failure corresponds to their lack.

Thieves, like "inbred" paupers, belong to the "blood-sucking" class. There is no injustice in ranking "inbred" paupers and thieves together. The voluntary pauper is merely a thief who has not the pluck of the man who lives by thieving. Both these classes are parasites of the most worthless description. Both of them live on the labours of persons who are no better armed by nature for the battle of life than they are. Many of those who pay the rates which feed and clothe the lusty beggars and paupers who will not work and the thieves who will steal, are persons in the feeblest of health, whose life is one long struggle against disease, weakness, and approaching death. Why should they add to their overwhelming toils in order that idle and vicious scoundrels may eat and sleep without labour? The instinct of a horse, or a dog, or a cat, prompts him to get rid of the parasites that torment him. Similarly, the instinct of the community makes us at least cry out when we feel ourselves smothered with the wretched human vermin which enjoys both home and food at our expense. That is a sound instinct. A state full of vigour and capacity does well to be impatient with "loafers" and "wasters." Of course the task of getting rid of a seriously burdensome social parasitism is not easy. Why should it be easy? Few things that are worth doing are easy. We have striven in this paper to put the full measure of their responsibility upon social parasites themselves. But all the responsibility is not theirs. If the community were in a vigorous condition of health, parasitism would rapidly diminish. It must be diminished; and it can only be reduced to a minimum by the energetic and discriminating action of the best mind of the community. Does any one expect social parasites to abolish themselves, or to turn themselves into industrious and deserving citizens? As well might we expect fleas and bugs to become sheep and oxen. The diminution or abolition of parasitism can only be the result of intelligent and strong action; and intelligent and strong action can only come from those who have intelligence and strength.

(*To be continued.*)

